# Role of Keap1-Nrf2/ARE signal transduction pathway in protection of dexmedetomidine preconditioning against myocardial ischemia/reperfusion injury

**DOI:** 10.1042/BSR20221306

**Published:** 2022-09-02

**Authors:** Hui-xian Li, Tai-hang Wang, Lin-xin Wu, Fu-shan Xue, Guo-hua Zhang, Tao Yan

**Affiliations:** 1 Department of Anesthesiology, National Cancer Center/National Clinical Research Center for Cancer/Cancer Hospital, Chinese Academy of Medical Sciences and Peking Union Medical College, Beijing 100021, China; 2 Department of Anesthesiology, Beijing Friendship Hospital, Capital Medical University, Beijing 100050, China; 3 Department of Anesthesiology, National Cancer Center/National Clinical Research Center for Cancer/Hebei Cancer Hospital, Chinese Academy of Medical Sciences, Langfang 065001, China

**Keywords:** Dexmedetomidine, Keap1-Nrf2/ARE signal transduction pathway, Myocardial ischemia/reperfusion injury, Oxidative stress

## Abstract

Objective: To explore the role and mechanism of the Kelch sample related protein-1-nuclear factor erythroid-2 related factor 2/antioxidant response element (Keap1-Nrf2/ARE) signaling pathway in protection of dexmedetomidine (DEX) preconditioning against myocardial ischemia/reperfusion injury (MIRI). Methods: A total of 70 male SD rats were randomly divided into seven equal groups (*n*=10): blank control (S group), ischemia/reperfusion injury (C group), DEX preconditioning (DEX group), tertiary butylhydroquinone (tBHQ) control (tBHQ group), combined tBHQ and DEX preconditioning (tBHQ+DEX group), all-trans retinoic acid (ATRA) control (ATRA group), and combined ATRA and DEX preconditioning (ATRA+DEX group). Serum creatine kinase-MB (CK-MB) and cardiac troponin I (cTnI) concentrations were measured by ELISA kits, and the infarct size (IS) was assessed by Evan’s blue and 2,3,5-triphenyltetrazolium chloride (TTC) staining. Oxidative stress was assessed through Western blotting for expression of Keap1-Nrf2/ARE pathway members and oxidative stress markers. Results: Cardioprotection of DEX, tBHQ, and tBHQ+DEX preconditioning treatments were shown as lower concentrations of serum CK-MB and cTnI and a smaller IS following MIRI in rats compared with those of MIRI rats without pre-treatment. In addition, tBHQ+DEX preconditioning exhibited stronger myocardial protection compared with DEX preconditioning. Mechanistically, the cardioprotection offered by DEX, tBHQ, and tBHQ+DEX preconditioning treatments was mediated via exerting antioxidant stress through activation of the Keap1-Nrf2/ARE signal transduction pathway. Conversely, the protective effects of DEX were diminished by blocking the Keap1-Nrf2/ARE pathway with inhibitor ATRA. Conclusion: DEX preconditioning protects against MIRI by exerting antioxidant stress through activation of the Keap1-Nrf2/ARE signal transduction pathway, while inhibition of the Keap1-Nrf2/ARE signal transduction pathway reverses the protective effect of DEX preconditioning on MIRI.

## Introduction

Myocardial ischemia/reperfusion injury (MIRI) is a phenomenon by which the process of myocardial reperfusion can further induce cardiac injury [1]. Oxidative stress is known to play an important role in MIRI [2]. In the ischemia/reperfusion procedure, the tissues are damaged due to ischemia and anoxia, which leads to sequent disorder of ATP synthesis and enhanced ATP catabolism with excessive reactive oxygen species (ROS) [3]. Excessive ROS not only directly act on myocardial cells, causing lipid peroxidation of cell membranes, DNA damage, and protein degeneration in cells but can also indirectly act as an intracellular messenger, activating various signaling pathways and resulting in tissue injury [2].

Epoxy chloropropane Kelch sample related protein-1 (Keap1)- nuclear factor erythroid-2 related factor 2 (Nrf2)/antioxidant response element (ARE) signal transduction pathway is one of the most significant pathways working against oxidative stress [4]. When oxidative stress occurs in myocardial tissue, the Keap1-Nrf2/ARE signal transduction pathway is activated with Nrf2 dissociating from Keap1. Nrf2 then translocates into the nucleus and combines with ARE in rapid sequence to stimulate expression of downstream antioxidative genes [5]. The pathway regulates transcription activities of phase II metabolic enzyme genes and antioxidative genes. NAD(P)H:quinone oxidoreductase-1 (NQO1) is one of the phase II metabolic enzymes regulated by Nrf2. NQO1 can prevent the production of ROS and has antioxidant effects [6]. Antioxidative proteins and enzymes that are regulated by Nrf2 predominantly include heme oxygenase-1 (HO-1), superoxide dismutase (SOD), γ-glutamylcysteine synthetase (γ-GCS), etc. HO-1 prohibits the dissociated ferroheme from participating in oxidative stress reactions. In addition, degradation products of HO-1 are able to suppress inflammation and dilate blood vessels, ultimately improving the blood circulation of tissues [7,8]. SOD and glutathione peroxidase (GSH-Px) are crucial enzymes with roles in the elimination of ROS, while malondialdehyde (MDA) is produced from lipid oxidation of the cell membrane. The concentration of each of SOD, MDA, and GSH-Px reflects the oxidative stress status [9]. The Keap1-Nrf2/ARE signal transduction pathway has a powerful impact in improving cardiac remodeling and cardiac dysfunction by regulating expression of multiple target genes [10].

As a highly selective activator of α2 adrenergic receptor, dexmedetomidine (DEX) mediates sedation, analgesia, and sympathetic inhibition and is therefore widely used in clinical anesthesiology and intensive care [11]. The protective effect of DEX against ischemia/reperfusion injury (IRI) is closely related to antioxidative-stress reactions. DEX was previously confirmed to protect against MIRI by ameliorating oxidative stress through the thioredoxin-1 (Trx1)-dependent Akt pathway and attenuating endothelial dysfunction in rats [12,13]. Karahan et al. [14] constructed a rat *in vivo* renal IRI model and showed that a single celiac injection of DEX (25 μg/kg) at 5 min before reperfusion restrained oxidative stress and therefore protected the rat kidneys from IRI. Meanwhile, Wang et al. [15] administered a continuous infusion of DEX before reperfusion and observed that DEX protected diabetic rats from lung ischemia/reperfusion injury by suppressing oxidative stress. Zeng et al. [16] investigated cerebral IRI in diabetic rats and concluded that a continuous infusion of DEX 30 min before ischemia reduced apoptosis of neurocytes and inhibited oxidative stress and inflammation. These studies clearly indicated that suppression of oxidative stress is an essential mechanism of IRI protection provided by DEX.

Since the Keap1-Nrf2/ARE pathway is instrumental in MIRI and oxidative stress, this study aimed to explore the efficiency of the Keap1-Nrf2/ARE pathway in cardioprotection of DEX preconditioning. Tertiary butylhydroquinone (tBHQ) or all-trans retinoic acid (ATRA), which work as a Keap1-Nrf2/ARE agonist or an inhibitor of Keap1-Nrf2/ARE, respectively, were utilized as interventions of DEX preconditioning.

## Materials and methods

### Animals

Seventy healthy male Sprague-Dawley rats, aged 8 weeks and weighing 200–230 g, were used in the research. The rats were provided by Beijing Weitonglihua Experimental Animal Technic Company Limited (SCXK 2014-0007). All rats were fed, and some were administered with tBHQ and ATRA. Rats were used for establishment of a MIRI model after 8 weeks.

### Establishment of rat *in vivo* MIRI model

The rat *in vivo* MIRI model was established according to a previous study [17]. Briefly, rats were anesthetized by intraperitoneal injection of 1% pentobarbital sodium (70 mg/kg) and endotracheally intubated for mechanical ventilation. A left thoracotomy was performed via the fourth intercostal space, and the left anterior wall and auricle of the heart were exposed. After pericardiotomy, a 5-0 silk ligature was placed under the left anterior descending (LAD) coronary artery. After an equilibration period of 10 min, the ligature was tied for 30 min to block blood flow of the LAD and then relaxed for 120 min to resume blood flow of the LAD, thus producing local MIRI.

### Experiment design

**Figure 1:**
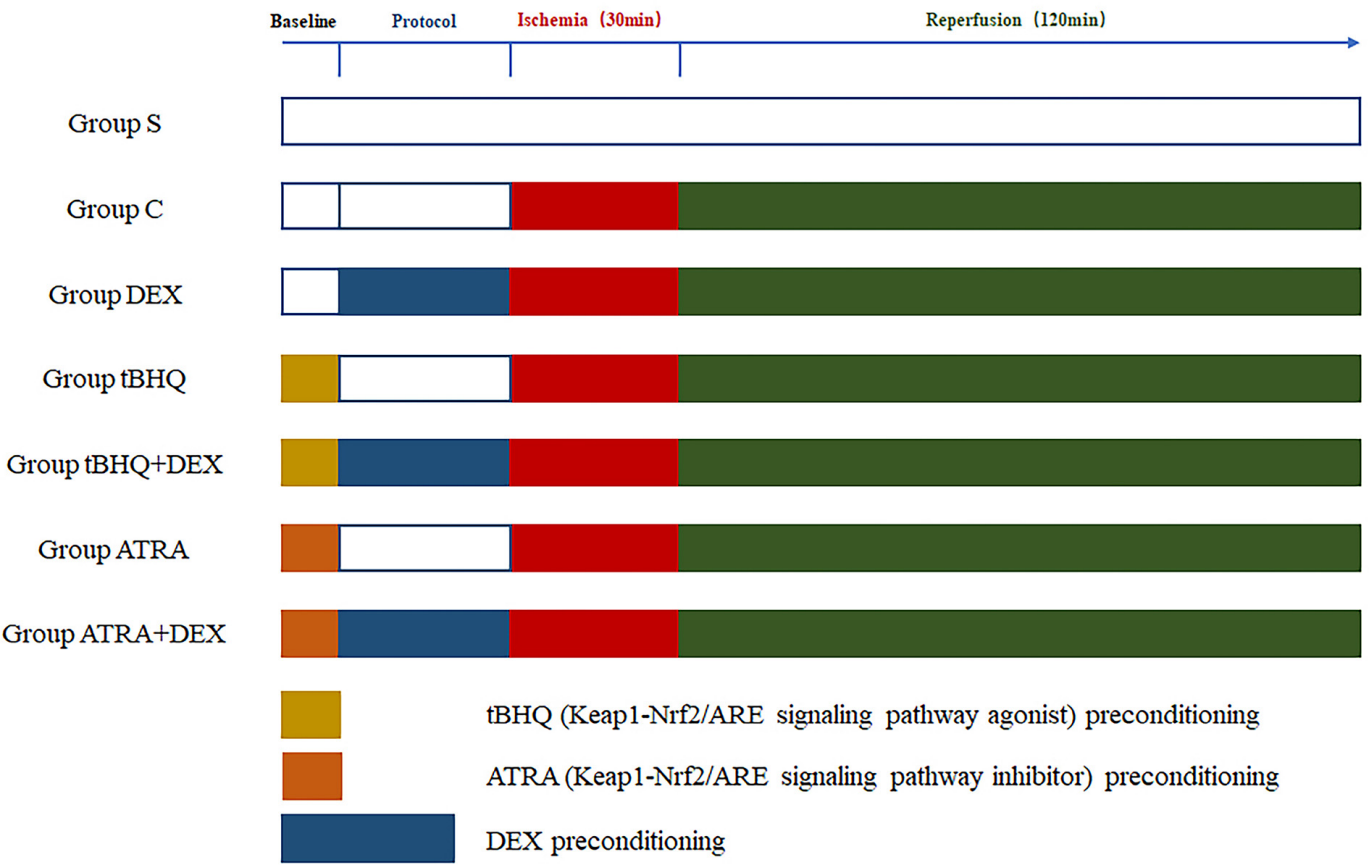
Experimental protocol of the groups Group S: Blank control group; Group C: IRI group; Group DEX: DEX preconditioning group; Group tBHQ: tBHQ control group; Group tBHQ+DEX: combined tBHQ and DEX preconditioning group; Group ATRA: ATRA control group; Group ATRA+DEX: combined ATRA and DEX preconditioning group.

A total of 70 rats were randomly divided into seven equal groups (*n*=10) by random number table and the protocol depicted in Figure 1. The groups were: (1) blank control (S group): a silk string was placed under LAD without ligation followed by 10 min equilibration and 180 min observation; (2) ischemia/reperfusion injury (C group): after the silk string placement and 10 min equilibration, normal saline was administered for 30 min followed by 30 min ischemia and 120 min reperfusion; (3) DEX preconditioning (DEX group): the dose of DEX was used according to a previous study [18], administration of DEX 6 μg/kg/h × 10 min + 0.7 μg/kg/h × 15 min followed by MIRI; (4) tBHQ control (tBHQ group): according to a previous study [19], the rats received a daily intraperitoneal injection of tBHQ (25 mg/kg) for 3 days before the experiment and the other operations were the same as C group; (5) DEX preconditioning with tBHQ (tBHQ+DEX group): the rats were intraperitoneally injected with tBHQ (25 mg/kg) for 3 days before the experiment and the other operations were the same as DEX group; (6) ATRA control (ATRA group): according to a previous study [20], the rats received intraperitoneal injection of ATRA (10 mg/kg) daily for 2 weeks before the experiment and the other operations were the same as C group; (7) DEX preconditioning with ATRA (ATRA+DEX group): the rats were intraperitoneally injected with ATRA (10 mg/kg) daily for 2 weeks before the experiment and the other operations were same as DEX group. Five rats in each group were used for measurements of infarct size (IS), and five were used for Western blotting and oxidative stress measurements.

### Observed variables

#### General conditions

According to a previous study, blood glucose concentration was measured by tail vein [21]. Rat weight and blood glucose concentration before anesthesia and central temperature after anesthesia were recorded.

#### Hemodynamics monitoring

The BL-420F biological function experiment system was used in the IRI procedure to continuously monitor heart rate (HR), systolic blood pressure (SBP), diastolic blood pressure (DBP), mean blood pressure (MBP), and lead II ECG of the rats. Hemodynamic data of a successfully established model that had equilibrated for 10 min were set as the base value. HR, SBP, and MBP were collected in the ischemia period (1, 5, 10, and 30 min) and reperfusion period (30, 60, and 120 min). The product of HR and SBP, which was the rate-pressure product (RPP), was taken as the cardio oxygen consumption index.

#### Arrythmia analysis and scoring

The peaks of arrhythmia score (AS) in the ischemia period and the first 30 min of the reperfusion period were recorded. By referring to previous studies [22], AS was graded as follows to quantify the severity of arrhythmia: 0, without arrhythmia; 1, <3 ventricular premature contractions (VPC) per min; 2, ≥3 VPC per min; 3, <3 episodes of ventricular tachycardia (VT) per min; 4, ≥3 episodes of VT per min or <3 episodes of transient fibrillation (VF) per min; 5, frequent (≥3 episodes per min) or sustained VF or death of the rat. The ratios of VT and VF were calculated.

#### Measurements of IS

One milliliter of Evan’s blue dye (20 mg/ml) was injected into the carotid artery after the reperfusion, and the rats were then killed. The hearts were removed and cut into five pieces of thickness following the direction from the LAD to the cardiac apex. The unstained area was the area at risk (AAR). The pieces of the heart were incubated in 10 mg/ml solution of 2,3,5-triphenyltetrazolium chloride (TTC) at 37°C for 5 min. The viable area stained red while the infarcted tissue stained characteristic white. Photographs of the samples were processed with Image-Pro Plus 5.0. The percentage of IS and AAR to total area was calculated. The IS was defined as percentage of infarction divided by percentage of AAR.

#### Measurements of serum CK-MB and cTnI

Samples of venous blood (2 ml per piece) from the right internal jugular veins at 120 min of reperfusion were collected in tubes containing EDTA. After being held static for 30 min, the samples were centrifugated at 301.9 ***g***. The resulting supernatant was collected and the serum concentrations of creatine kinase-MB (CK-MB) and cardiac troponin I (cTnI) were assessed, respectively, using enzyme-linked immunosorbent assay (ELISA) kits specifically for rats, following the steps of the manufacturer’s instructions (Elabscience, Wuhan, China).

#### Myocardial expression of Nrf2, HO-1, and NAD(P)H: quinone oxidoreductase 1 (NQO1) by Western blotting

Myocardial tissues in the ischemic area of five rats were collected after reperfusion and stored at −20°C, and expression of nucleus protein Nrf2, total protein HO-1, and total protein NQO1 were tested and assessed by Western blotting. The tissues were cut into small pieces, and protein lysis buffer (P001, Beijing, China) was used to extract proteins. The protein concentration was then measured by bicinchoninic acid (BCA) assay. Equal amounts of protein (50 μg) from each group were electrophoresed by SDS-PAGE (15% separation gel, 5% concentration gel), and the separated proteins were transferred to nitrocellulose membrane. After blocking and washing, rabbit Nrf2 polyclonal antibody (1:2000, ab137550, Abcam, U.S.A.), rabbit HO-1 monoclonal antibody (1:2000, ab189491, Abcam), rabbit NOQ1 polyclonal antibody (1:1000, ab34173, Abcam), rabbit Histone H3 polyclonal antibody (1:5000, ab1791, Abcam), and rabbit monoclonal antibodies GAPDH (1:1000, 5174, CST, USA) were added, respectively, to each accordant sample and the samples were incubated overnight at 4°C with shaking. Subsequently, the membranes were incubated with horseradish peroxidase-conjugated secondary antibody (1:10,000) for 1 h at room temperature. Enhanced chemiluminescence was used to visualize the antigen–antibody complex in the membranes. Density of the bands on the membranes was assessed and quantified with ImageJ 1.6. Finally, values of relative protein expression were acquired by standardizing the band density of Nrf2 with Histone H3, and the gray levels of HO-1 and NQO1 with GAPDH.

#### Marker of oxidative stress measurements

Tissues from the infarction area of five rats were collected, and SOD activity within the tissues was assayed using a commercial kit (A001-1-1, Nanjing, China). The degree of lipid peroxidation was evaluated by MDA kit (A003-1, Nanjing, China), and GSH-Px levels were tested with 5,5′-dithiobis-(2-nitrobenzoic acid) (DTNB) using a commercial kit (A005, Nanjing, China).

### Statistical analysis

Measurement data of normal distribution were expressed as mean ± standard deviation 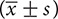, and their homogeneity of variance was tested by the Levene median test. For normally distributed data with homogeneous variance, a one-way analysis of variance (one-way ANOVA) was adopted and the Student–Newman–Keuls test was performed in close sequence to achieve intergroup comparisons. For data that were not normally distributed or had inhomogeneous variance, the Kruskal–Wallis test was used to compare one group with another. The enumeration data were expressed as percentages and cases and were compared using χ2 test or Fisher’s Exact Test. *P*<0.05 was considered statistically significant. All data analysis was accomplished with SPSS (version 19.0, SPSS, Inc., Chicago, IL, U.S.A.).

## Results

### General conditions and hemodynamics

As summarized in Table 1, there were no significant differences in body weight, temperature, and blood glucose concentration of the rats among all seven groups (*P*>0.05). The changes of HR, MAP, and RPP of rats are recorded in Table 2. The initial HR, MAP, and RPP among all rats showed no significant difference (*P*>0.05). At 1 min of ischemia, the HR in DEX group, tBHQ+DEX group, and ATRA+DEX group decreased significantly (*P*<0.05) compared with accordant initial levels. The MAP and RPP in all the groups except S group decreased significantly (*P*<0.05). During the ischemia period, a significant decrease of RPP in DEX group, tBHQ+DEX group, and ATRA+DEX group was detected (*P*<0.05). During the reperfusion period, the HR, MAP, and RPP of rats in all groups recovered gradually.

**Table 1:** General conditions of each group 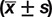

Groups (*n*=10)	Weight (g)	Temperature (°C)	Blood glucose (mmol/L)
**S**	335.8 ± 13.4	37.4 ± 0.2	4.0 ± 0.4
**C**	331.7 ± 16.5	37.3 ± 0.2	5.3 ± 0.8
**DEX**	328.2 ± 12.9	37.4 ± 0.2	5.6 ± 0.6
**tBHQ**	330.8 ± 16.6	37.3 ± 0.1	5.1 ± 0.6
**tBHQ+DEX**	334.7 ± 11.4	37.3 ± 0.2	5.4 ± 0.7
**ATRA**	333.0 ± 18.9	37.4 ± 0.2	5.1 ± 0.6
**ATRA+DEX**	334.8 ± 12.2	37.5 ± 0.2	5.2 ± 0.9

Group ATRA: ATRA control group; Group ATRA+DEX: combined ATRA and DEX preconditioning group; Group C: IRI group; Group DEX: DEX preconditioning group; Group S: Blank control group; Group tBHQ: tBHQ control group; Group tBHQ+DEX: combined tBHQ and DEX preconditioning group. All the data were expressed as mean ± standard deviation 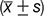, *n*=10.

**Table 2:** Hemodynamic changes 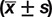

Groups	Basic level	Ischemia period				Reperfusion period		
		1 min	5 min	15 min	30 min	30 min	60 min	120 min
**HR (bmp)**								
**S**	384.0 ± 24.0	390.5 ± 25.8	380.0 ± 14.4	381.0 ± 20.2	375.5 ± 16.2	369.3 ± 22.2	368.0 ± 21.3	366.6 ± 20.4
**C**	396.5 ± 38.6	413.5 ± 45.6	378.0 ± 76.9	383.3 ± 34.2	356.1 ± 76.6	349.3 ± 37.1*	356.0 ± 36.9	371.3 ± 23.1
**DEX**	385.6 ± 35.9	335.5 ± 17.6*	317.8 ± 16.7*	323.0 ± 29.4*	326.0 ± 20.7*	369.8 ± 27.5	361.7 ± 21.9	357.7 ± 24.2
**tBHQ**	394.5 ± 29.4	418.8 ± 39.7	373.1 ± 30.6	345.5 ± 37.4	360.1 ± 28.0	362.3 ± 26.1	368.3 ± 39.3	356.8 ± 36.8
**tBHQ+DEX**	395.8 ± 42.1	336.3 ± 32.9*	335.3 ± 27.7*	327.0 ± 45.4*	336.8 ± 13.2*	348.8 ± 24.7*	353.0 ± 32.9	363.1 ± 54.6
**ATRA**	391.3 ± 35.2	409.7 ± 21.6	380.1 ± 30.7	373.1 ± 35.9	352.1 ± 16.1	377.5 ± 15.1	373.1 ± 28.4	375.6 ± 42.10
**ATRA+DEX**	392.5 ± 48.7	335.8 ± 49.0*	333.8 ± 32.6*	336.0 ± 34.1*	333.6 ± 54.6*	345.5 ± 38.3*	366.6 ± 53.3	363.3 ± 72.4
**MAP (mmHg)**								
**S**	120.3 ± 9.9	123.8 ± 3.6	120.3 ± 13.9	123.1 ± 6.3	123.0 ± 10.2	127.8 ± 5.2	122.5 ± 10.3	123.8 ± 7.1
**C**	121.7 ± 22.3	81.8 ± 17.3*	88.3 ± 19.7*	89.3 ± 18.3*	88.0 ± 18.0	92.0 ± 17.5	84.8 ± 11.0*	86.6 ± 17.4*
**DEX**	119.3 ± 11.7	65.5 ± 19.8*	66.3 ± 14.9*	67.8 ± 19.8*	69.0 ± 14.9*	88.8 ± 14.0	91.3 ± 12.4	95.8 ± 15.0
**tBHQ**	122.1 ± 11.1	68.3 ± 21.4*	68.7 ± 24.7*	83.0 ± 26.6*	79.0 ± 29.3*	93.5 ± 18.9	98.8 ± 15.9	98.6 ± 16.3
**tBHQ+DEX**	121.6 ± 12.7	67.1 ± 4.5*	69.5 ± 14.6*	70.8 ± 10.7*	68.1 ± 4.7*	81.5 ± 22.2*	80.1 ± 20.6*	82.1 ± 25.7*
**ATRA**	120.3 ± 5.4	65.5 ± 19.6*	78.3 ± 10.2*	78.1 ± 24.2*	77.1 ± 19.5*	90.1 ± 20.0	89.7 ± 15.0	100.0 ± 14.4
**ATRA+DEX**	121.8 ± 26.1	67.8 ± 18.3*	73.0 ± 25.4*	75.0 ± 16.4*	74.8 ± 19.0*	88.8 ± 29.0	90.7 ± 34.6	90.8 ± 36.6
**RPP (1000/min/mm Hg)**								
**S**	50.0 ± 7.9	52.2 ± 4.1	49.5 ± 6.4	50.7 ± 5.1	49.9 ± 5.6	50.9 ± 4.6	48.8 ± 6.5	49.1 ± 4.6
**C**	52.2 ± 12.7	38.0 ± 7.2	37.2 ± 10.2	38.1 ± 5.8	38.5 ± 10.8	35.6 ± 8.0	31.9 ± 4.1*	35.9 ± 7.6
**DEX**	49.9 ± 4.7	25.3 ± 7.9*	24.2 ± 4.6*	25.1 ± 7.9*	25.8 ± 7.6*	36.5 ± 7.3	35.0 ± 5.3	37.8 ± 7.6
**tBHQ**	52.1 ± 8.6	36.8 ± 5.7	36.4 ± 6.7	37.7 ± 9.7	35.0 ± 7.5	37.1 ± 6.8	38.4 ± 7.1	36.2 ± 7.8
**tBHQ+DEX**	52.1 ± 5.6	28.2 ± 2.8*	29.0 ± 6.9*	28.0 ± 6.7*	27.1 ± 5.7*	32.1 ± 8.7*	34.1 ± 9.7*	35.4 ± 9.0
**ATRA**	51.0 ± 12.3	34.9 ± 7.3	36.4 ± 3.4	36.9 ± 4.5	35.7 ± 6.5	37.8 ± 7.7	37.2 ± 7.9	41.3 ± 12.4
**ATRA+DEX**	51.7 ± 3.3	27.1 ± 10.7*	28.5 ± 11.2*	29.4 ± 5.4*	28.3 ± 4.5*	32.1 ± 7.9 ± 14.1*	35.9 ± 7.5	36.6 ± 6.9

Group ATRA: ATRA control group; Group ATRA+DEX: combined ATRA and DEX preconditioning group; Group C: IRI group; Group DEX: DEX preconditioning group; Group S: Blank control group; Group tBHQ: tBHQ control group; Group tBHQ+DEX: combined tBHQ and DEX preconditioning group. All the data were expressed as mean ± standard deviation 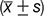, *n*=10; *P<0.05: compared with basic level.

### Arrythmia analysis and scoring

**Figure 2:**
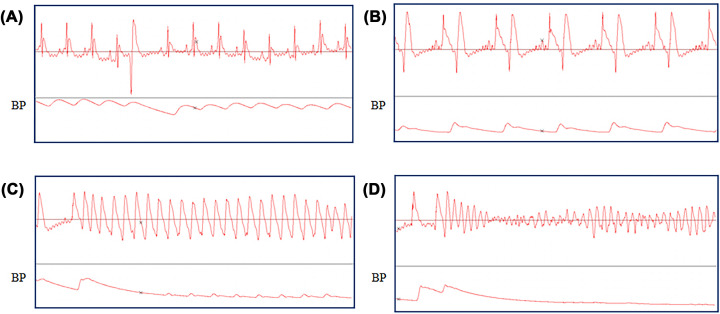
Typical arrhythmia changes The electrocardiogram of VPC (**A**), ventricular bigeminy (**B**), VT (**C**), and ventricular fibrillation (**D**).

Typical arrhythmias are shown in Figure 2, and arrythmia analysis and scoring of all groups are recorded in Table 3. During 5–15 min of ischemia, rats of all groups except S group exhibited quantities of multifocal VPC and VT whose frequency and duration varied. Some rats even had VF. After 15 min of ischemia, the frequency and duration of ventricular arrythmia lowered but VF was still detectable in some of the rats. During the ischemia period, the number of rats that had VT or VF (or both) in each group and the ventricular AS of the rats showed no significant differences (*P*>0.05). In the reperfusion period, several rats exhibited VT with different frequency and duration but VF was not detected in any rat. Compared with C group, the number of rats with VT or VF in DEX group, tBHQ group, and tBHQ+DEX group decreased significantly (*P*<0.05). Compared with DEX group and tBHQ group, the number of rats with VT or VF in tBHQ+DEX group decreased significantly (*P*<0.05).

**Table 3:** Occurrence and scores of ventricular arrhythmias during the ischemia/reperfusion process

Groups (*n*=10)	Ischemia	Early stage of reperfusion	Ventricular arrythmia scoring	
	VT,VF	VT,VF	Ischemia	Early stage of reperfusion
	Animal numbers with VT and VF (Rate of occurring)		Median (Interquartile range)	
**S**	0	0	0 (0)	0 (0)
**C**	10 (100%)§	10 (100%)§	4 (1)§	4 (0.25)§
**DEX**	8 (80%)§	7(70%)^*§^	4 (1.25)§	3 (2)§
**tBHQ**	9 (90%)§	6 (60%)^*^§	4 (1.25)§	3 (1)§
**tBHQ+DEX**	8 (80%)§	3 (30%)^*†^^‡§^	3 (0.75)§	2 (2)§
**ATRA**	9 (90%)§	9 (90%)§	4 (0.75)§	3 (2)§
**ATRA+DEX**	10 (100%)§	10 (100%)§	4 (0.25)§	3 (1)§

Group ATRA: ATRA control group; Group ATRA+DEX: combined ATRA and DEX preconditioning group; Group C: IRI group; Group DEX: DEX preconditioning group; Group S: Blank control group; Group tBHQ: tBHQ control group; Group tBHQ+DEX: combined tBHQ and DEX preconditioning group. Data are expressed as cases, percentage, median and interquartile range, *n*=10; ^*^P<0.05: compared with group C; ^†^*P*<0.05: compared with group DEX; ‡*P*<0.05: compared with group tBHQ; §*P*<0.05: compared with group S.

### Infarct size

**Figure 3:**
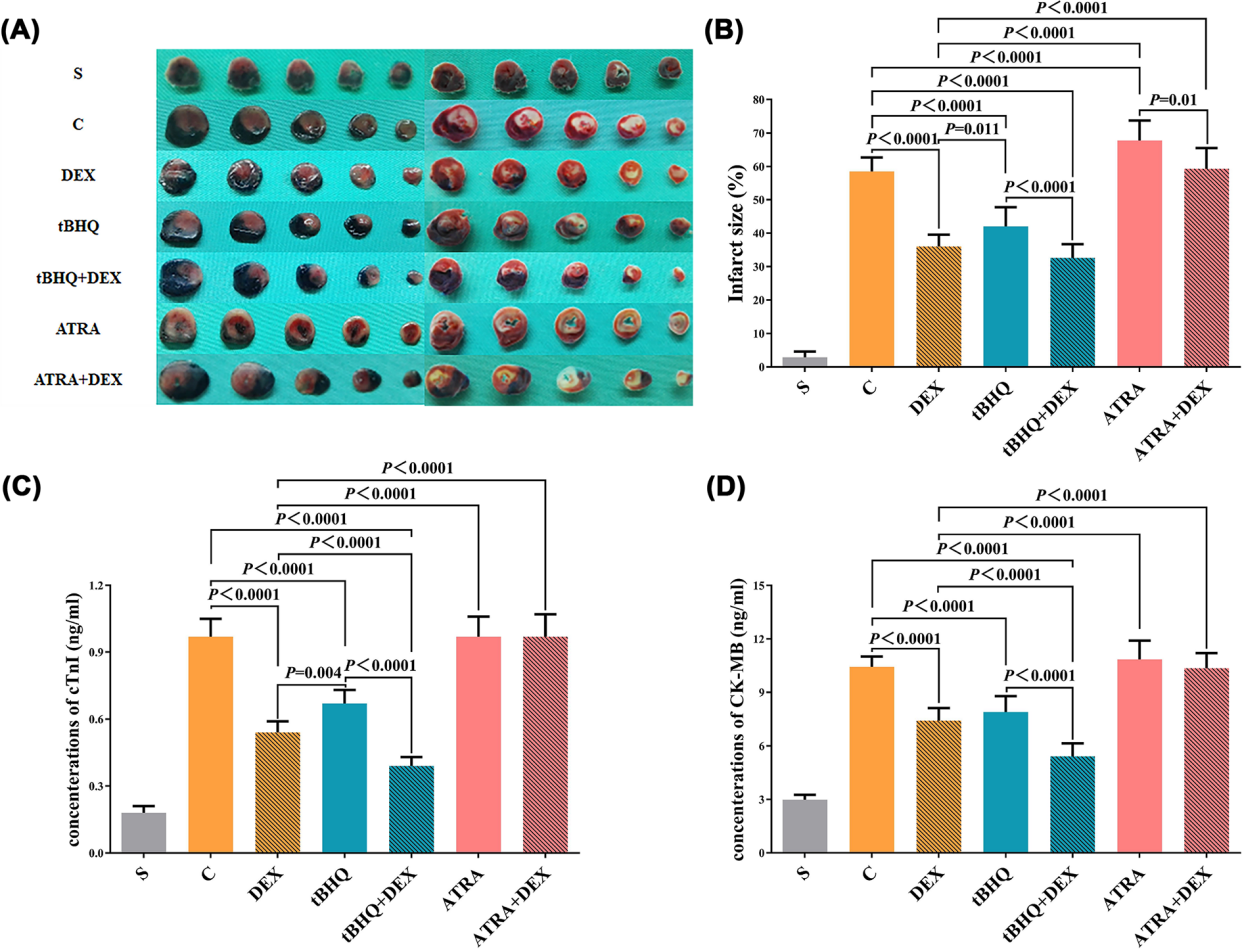
IS, serum CK-MB, and cTnI concentrations in the seven groups Typical stained myocardial slices (**A**), infarct size (**B**), and serum cTnI (**C**) and CK-MB (**D**) concentrations in the blank control (S), IRI (C), DEX preconditioning (DEX), tBHQ control (tBHQ), combined tBHQ and DEX preconditioning (tBHQ+DEX), ATRA control (ATRA), and combined ATRA and DEX preconditioning (ATRA+DEX) groups. All data were expressed as mean ± standard deviation 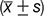, *n*=5.

Typical myocardial sections double-stained by Evan’s blue and TTC are shown in Figure 3A. AAR, IS, and their comparison among groups are summarized in Table 4 and Figure 3B. No significant difference was found in the ARR of each pair of groups (*P*>0.05). Compared with S group, the IS of the other six groups increased significantly (*P*<0.05). Compared with C group, the IS of DEX group, tBHQ group, and tBHQ+DEX group decreased significantly (*P*<0.05), ATRA group increased significantly (*P<0.*05). Compared with DEX group, the IS in tBHQ group, ATRA group, and ATRA+DEX group increased significantly (*P*<0.05). Furthermore, the IS in tBHQ+DEX group was decreased compared with that in tBHQ group (*P*<0.05), and the IS in ATRA+DEX group was markedly lower compared with that in ATRA group (*P*<0.05).

**Table 4:** Comparisons of AAR and IS 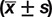

Groups (*n*=5)	AAR (%)	IS (%)
**S**	53.1 ± 3.5	2.9 ± 1.7
**C**	54.2 ± 4.6	58.5 ± 4.2*
**DEX**	52.8 ± 5.5	36.0 ± 3.5*^†^
**tBHQ**	54.5 ± 6.7	42.0 ± 5.7*^†‡^
**tBHQ+DEX**	51.6 ± 4.9	32.6 ± 4.1*^†§^
**ATRA**	56.5 ± 6.1	67.8 ± 5.9*^†‡^
**ATRA+DEX**	54.3 ± 4.2	59.3 ± 6.2*‡^║^

Group ATRA: ATRA control group; Group ATRA+DEX: combined ATRA and DEX preconditioning group; Group C: IRI group; Group DEX: DEX preconditioning group; Group S: Blank control group; Group tBHQ: tBHQ control group; Group tBHQ+DEX: combined tBHQ and DEX preconditioning group. All the data were expressed as mean ± standard deviation 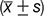, *n*=5; *P<0.05: compared with group S; ^†^*P*<0.05: compared with group C; ^‡^*P*<0.05: compared with group DEX; ^§^*P*<0.05: compared with group tBHQ; ^║^*P*<0.05: compared with group ATRA.

### Serum CK-MB and cTnI concentrations

Serum CK-MB and cTnI concentrations and comparison among groups are summarized in Figure 3C,D. Compared with S group, serum concentrations of CK-MB and cTnI in the other six groups increased significantly (*P*<0.05). Compared with C group, serum concentrations of CK-MB and cTnI in DEX group, tBHQ group, and tBHQ+DEX group decreased significantly (*P*<0.05). Compared with DEX group, serum concentrations of CK-MB and cTnI in tBHQ+DEX group decreased significantly (*P*<0.05) and those in ATRA group and ATRA+DEX group increased significantly (*P*<0.05). Compared with tBHQ group, serum concentrations of CK-MB and cTnI in tBHQ+DEX group decreased significantly (*P*<0.05).

### Expression of Keap1-Nrf2/ARE signal transduction pathway-associated proteins

**Figure 4:**
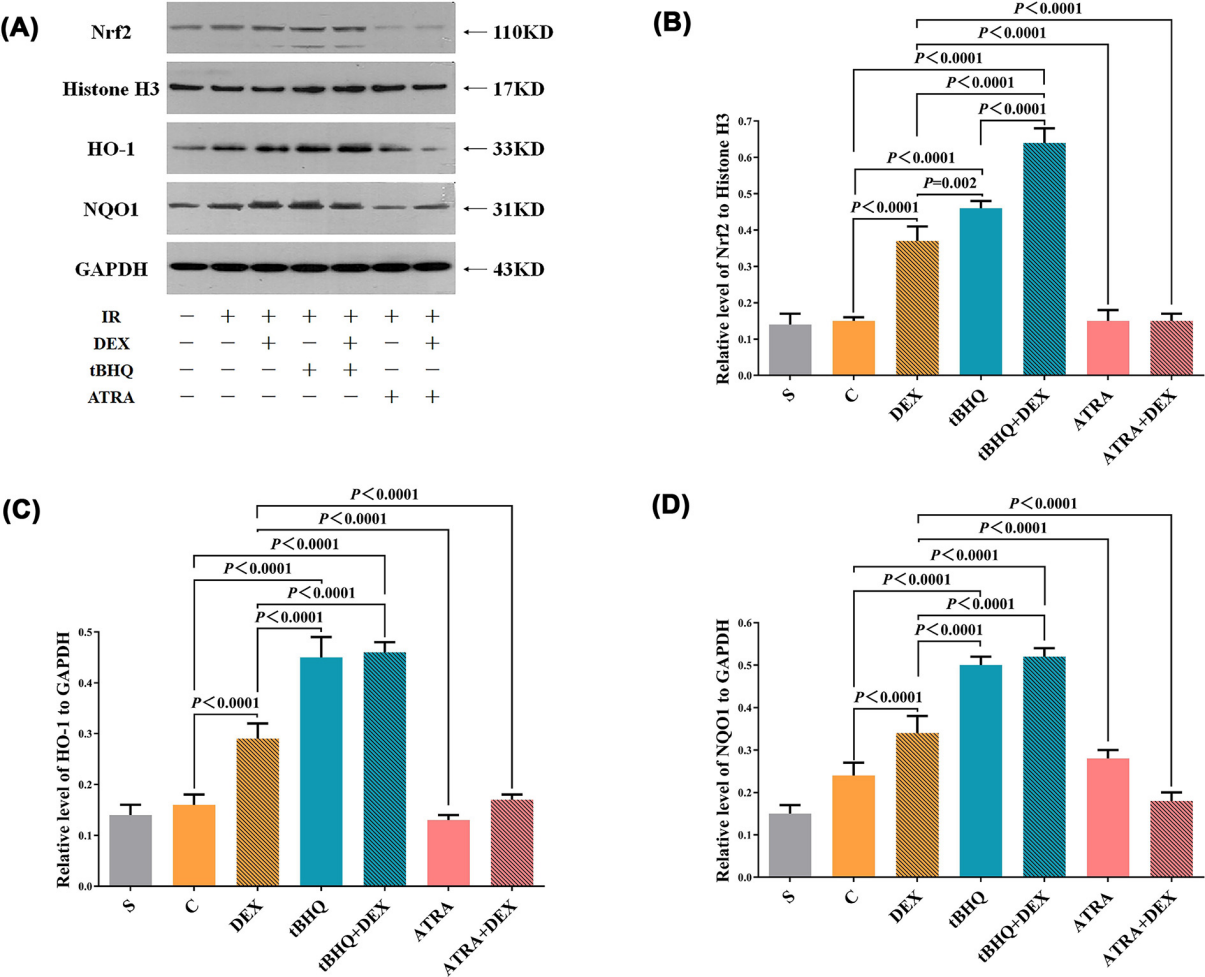
Western blotting for myocardial expression of Nrf2, HO-1, and NQO1 Western blots for myocardial expression of Nrf2, HO-1, and NQO1 (**A**), quantitative analysis for myocardial expression of Nrf2 (**B**), HO-1 (**C**), and NQO1 (**D**) in the blank control (S), IRI (C), DEX preconditioning (DEX), tBHQ control (tBHQ), combined tBHQ and DEX preconditioning (tBHQ+DEX), ATRA control (ATRA), and combined ATRA and DEX preconditioning (ATRA+DEX) groups. All data were expressed as mean ± standard deviation 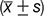, *n*=5.

Electrophoresis results of Nrf2 nucleus protein, HO-1 total protein, and NQO1 total protein in ischemia heart tissues are depicted in Figure 4A. Band density analysis of electrophoresis is shown in Figure 4B–D. Compared with S group, the expressions of Nrf2 nucleus protein, HO-1 total protein, and NQO1 total protein in ischemia heart tissues of DEX group, tBHQ group, and tBHQ+DEX group increased significantly (*P*<0.05) and were also slightly higher compared with those of C group (*P*>0.05). Compared with DEX group, myocardial expression of Nrf2, HO-1, and NQO1 in tBHQ group and tBHQ+DEX group increased significantly (*P*<0.05) and those in ATRA group and ATRA+DEX group decreased significantly (*P*<0.05). Compared with tBHQ group, myocardial Nrf2 expression in tBHQ+DEX group increased significantly (*P*<0.05).

### Levels of oxidative stress markers

**Figure 5:**
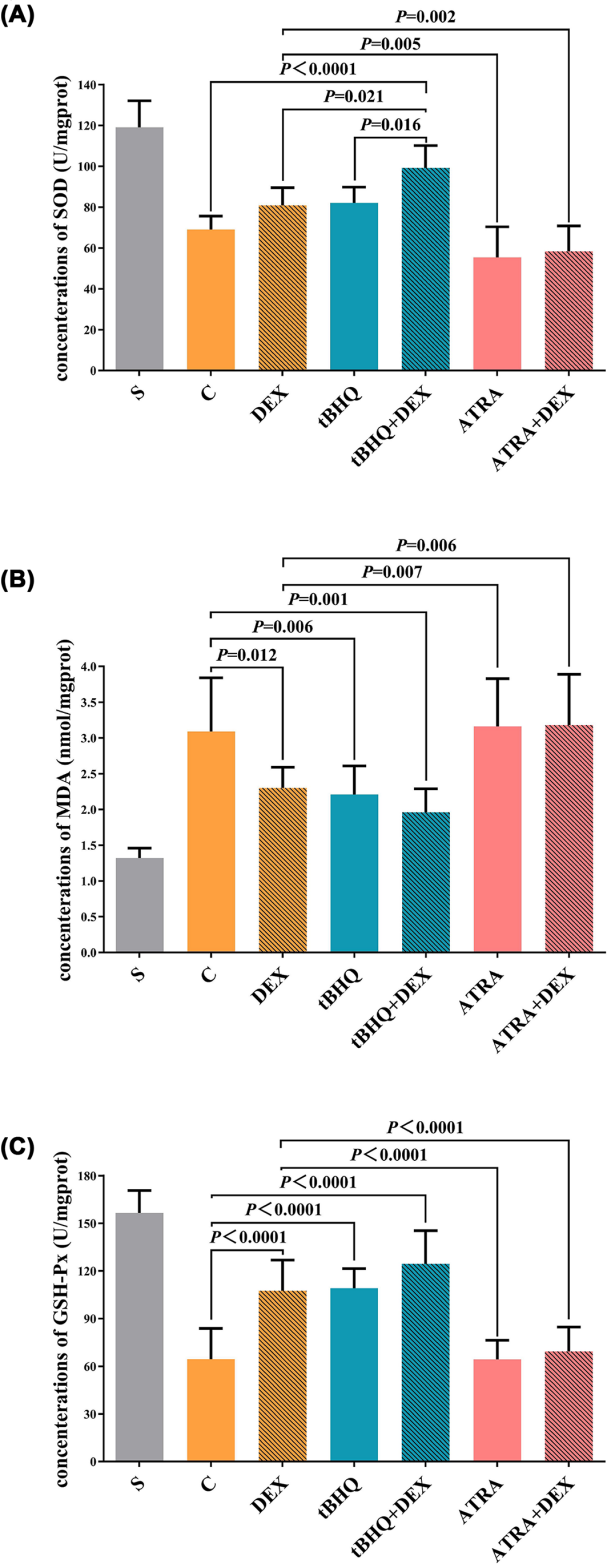
Levels of oxidative stress markers SOD (**A**), MDA (**B**), and GSH-Px (**C**) levels in the blank control (S), IRI (C), DEX preconditioning (DEX), tBHQ control (tBHQ), combined tBHQ and DEX preconditioning (tBHQ+DEX), ATRA control (ATRA), and combined ATRA and DEX preconditioning (ATRA+DEX) groups. All data were expressed as mean ± standard deviation 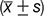, *n*=5.

SOD, MDA, and GSH-Px levels in ischemia heart tissues and comparisons among groups were recorded and are shown in Figure 5A–C. Compared with S group, myocardial SOD and GSH-Px levels in the other six groups decreased significantly (*P*<0.05) but MDA levels were increased (*P*<0.05). Compared with C group, SOD levels in tBHQ+DEX group increased significantly (*P*<0.05); in DEX group, tBHQ group, and tBHQ+DEX group, GSH-Px levels increased significantly but MDA levels lowered (both *P*<0.05). Compared with DEX group, myocardial SOD and GSH-Px levels in tBHQ+DEX group increased significantly (*P*<0.05), while in ATRA group and ATRA+DEX group, myocardial SOD and GSH-Px levels were markedly lower compared with those in DEX group yet MDA levels increased significantly (both *P*<0.05). Compared with tBHQ group, myocardial SOD levels in tBHQ+DEX group were significantly increased (*P*<0.05).

## Discussion

MIRI is a phenomenon whereby the process of myocardial reperfusion causes more injury compared with simple ischemia [1]. DEX is a highly selective α2-adrenergic receptor activator that has sedative, analgesic, and sympathetic inhibition effects [11]. DEX, therefore, has the potential to be used for reducing myocardial injury in the future. The Keap1-Nrf2/ARE signal transduction pathway is an essential antioxidative stress pathway that has a powerful impact in improving cardiac remodeling and cardiac dysfunction [10]. Thus, in the present study, it was hypothesized that DEX could affect MIRI by regulating the signal transduction pathway. A rat MIRI model was established in the present study, based on previous reportss, and an agonist (tBHQ) and an inhibitor (ATRA) of the Keap1-Nrf2/ARE pathway were used to disrupt DEX preconditioning. tBHQ facilitates Nrf2 transfer to the nucleus and sequentially stimulates the expression of phase II metabolic enzymes and antioxidation enzymes regulated by ARE [23]. On the contrary, inhibitor ATRA functions by restraining the activity of ARE [20].

Compared with C group, the expression levels of Nrf2 nuclear protein, HO-1, and NQO1 total protein in DEX group, tBHQ group, and tBHQ+DEX group increased significantly. SOD and GHS-Px levels were also increased, while the MDA level was decreased accordantly. Meanwhile the IS reduced and myocardial enzymes changed. Collectively, these observations indicated that DEX preconditioning could stimulate the Keap1-Nrf2/ARE signal transduction pathway, resist MIRI oxidative stress, and ultimately provide protection against MIRI. Erkens et al. [24] studied wild-type and Nrf2 gene knockout rats and discovered that Nrf2 gene knockout rats exhibited cardiovascular disorders, such as left ventricular diastolic dysfunction. Xu et al. [25] likewise used Nrf2 gene knockout rats and found out that after 30 min of LAD ligation, the IS of these rats doubled compared with wild-type rats. Aside from tBHQ, resveratrol is another Keap1-Nrf2/ARE signaling transduction pathway agonist [26]. Resveratrol improved cardiac function and reduced IS by stimulating the Keap1-Nrf2/ARE pathway [27]. Yu et al. [28] conducted a study on *in situ* liver transplantation in rats and proved that DEX could stimulate the Keap1-Nrf2/ARE pathway and regulate expression of GSH-Px, SOD, and MDA, and therefore prevented oxidative stress and alleviated renal injury in the rats during liver transplanting. The above studies and the current study reached the same conclusion that DEX intervention reduced oxidative stress by activating the Keap1-Nrf2/ARE signaling transduction pathway and providing protection against IRI. Furthermore, compared with DEX group or tBHQ group, IS and serum myocardial enzyme levels in tBHQ+DEX group decreased, indicating that the combination of DEX and agonist tBHQ may enhance MIRI protection.

When ATRA, the Keap1-Nrf2/ARE pathway inhibitor, was applied alone (ATRA group), expression of Nrf2, HO-1, and NQO1 decreased compared with C group. Accordantly, SOD and GSH-Px levels in ATRA group decreased yet MDA levels were elevated. Considering that IS and serum myocardial enzyme levels increased in ATRA group along with the changes above, it was concluded that blocking the anti-oxidation pathway without any protecting intervention could aggravate MIRI. When combining ATRA and DEX, no significant difference was found between IS of ATRA+DEX group and C group. This indicated that the myocardial protection of DEX preconditioning disappeared once the Keap1-Nrf2/ARE signaling pathway was blocked. IS in ATRA+DEX group decreased markedly compared with that in ATRA group. This might be due to various reasons. First, DEX preconditioning was still partially efficient in myocardial protection even though the Keap1-Nrf2/ARE pathway was blocked. Previous studies indicated that DEX realized its MIRI protection through the PI3K/AKT pathway and eNOS/NO pathway, which interacted with the Keap1-Nrf2/ARE pathway [29,30]. After analyzing SOD, MDA, and GSH-Px data in the current study, it cannot be excluded that DEX preconditioning still functioned against inflammation and apoptosis even though its antioxidation effect was eliminated by Keap1-Nrf2/ARE inhibitors [18,31]. Second, ATRA is an unselective Nrf2 inhibitor and may not be able to completely block the pathway [32]. In 2015, Wu [33] studied lung IRI and discovered that ATRA suppressed the antioxidation effect of remote ischemia preconditioning by blocking the Keap1-Nrf2/ARE pathway and ultimately reversed the lung IRI protection provided by remote ischemia preconditioning. Taking the current study into consideration, ATRA efficiently blocked the Keap1-Nrf2/ARE pathway and reversed myocardial protection of the intervention treatments. In summary, previous research and the results of the present study demonstrate that ATRA can effectively block the Keap1-Nrf2/ARE pathway and reverse the myocardial protective effect of DEX preconditioning.

There are limitations in the current study and previous research. First, utilization of DEX varied in studies on MIRI; for instance, single-dose celiac injection preconditioning, intravenous pump injection preconditioning, and single-dose intravenous injection preconditioning were all reported [10,28]. MIRI protection changed with the methods of delivery and dose of DEX. Due to the limitation of time and research scale, only single-dose pump injection was used in the current study without an investigation of dose gradients. Second, the present study referred to previous research to formulate intervention time and dose of tBHQ and ATRA [11,12]. However, tBHQ and ATRA are not specific agonist and inhibitor, respectively, of the Keap1-Nrf2/ARE pathway, which may affect the intervention of the signaling pathway [32,34]. Inhibition of the Nrf2 pathway was recently realized through other processes aside from ATRA intervention, for example, the Nrf2 gene knockout. In the future, relevant animal models will be established.

In conclusion, DEX preconditioning or Keap1-Nrf2/ARE pathway agonist tBHQ protect against MIRI by exerting antioxidant stress through activation of the Keap1-Nrf2/ARE signal transduction pathway. The combined application of DEX preconditioning and agonist tBHQ can enhance the protective effect against MIRI. However, blocking the Keap1-Nrf2/ARE signal transduction pathway with ATRA may reverse the protective effect of DEX preconditioning on MIRI.

## Data Availability

All data of this research will be available and accessible. Please contact the corresponding author for data requests.

## Abbreviations

γ-GCS: γ-glutamylcysteine synthetase
AAR: area at risk
ARE: antioxidant response element
AS: arrhythmia score
ATRA: all-trans retinoic acid
CK-MB: creatine kinase-MB
cTnI: cardiac troponin I
DBP: diastolic blood pressure
DEX: dexmedetomidine
GSH-Px: glutathione peroxidase
HO-1: heme oxygenase-1
HR: heart rate
IRI: ischemia/reperfusion injury
IS: infarct size
Keap1: Epoxy chloropropane Kelch sample related protein-1
LAD: left anterior descending coronary artery
MBP: mean blood pressure
MDA: malondialdehyde
MIRI: myocardial ischemia/reperfusion injury
NQO1: NAD(P)H:quinone oxidoreductase-1
Nrf2: nuclear factor erythroid-2 related factor 2
ROS: reactive oxygen species
RPP: rate-pressure product
SBP: systolic blood pressure
SOD: superoxide dismutase
tBHQ: tertiary butylhydroquinone
Trx1: thioredoxin-1
TTC: 2,3,5-triphenyltetrazolium chloride
VF: transient fibrillation
VPC: ventricular premature contractions
VT: ventricular tachycardia
